# Research on the Potential of Spherical Triboelectric Nanogenerator for Collecting Vibration Energy and Measuring Vibration

**DOI:** 10.3390/s20041063

**Published:** 2020-02-15

**Authors:** Chuan Wu, He Huang, Rui Li, Chenxing Fan

**Affiliations:** 1 School of Mechanical and Electronic Information, China University of Geosciences (Wuhan), Wuhan 430074, China; wuchuan@cug.edu.cn (C.W.); fancx@cug.edu.cn (C.F.); 2 Powerchina Hubei Electric Engineering Corporation Limited, Wuhan 430040, China; huangh92-huby@powerchina.cn; 3 State Key Laboratory of Coal Mine Disaster Dynamics and Control, Chongqing University, Chongqing 400044, China

**Keywords:** triboelectric nanogenerator, self-powered, energy harvesting, vibration sensor

## Abstract

The traditional downhole drilling vibration measurement methods which use cable or battery as power supplies increase the drilling costs and reduce the drilling efficiency. This paper proposes a spherical triboelectric nanogenerator, which shows the potential to collect the downhole vibration energy and measure the vibration frequency in a self-powered model. The power generation tests show that the output signal amplitude of the spherical triboelectric nanogenerator increases as the vibration frequency increases, and it can reach a maximum output voltage of 70 V, a maximum current of 3.3 × 10^−5^ A, and a maximum power of 10.9 × 10^−9^ W at 8 Hz when a 10-ohm resistor is connected. Therefore, if the power generation is stored for a certain period of time when numbers of the spherical triboelectric nanogenerators are connected in parallel, it may provide intermittent power for the low-power downhole measurement instruments. In addition, the sensing tests show that the measurement range is 0 to 8 Hz, the test error is less than 2%, the applicable working environment temperature is below 100 degrees Celsius, and the installation distance between the spherical triboelectric nanogenerator and the vibration source should be less than the critical value of 150 cm because the output signal amplitude is inversely proportional to the distance.

## 1. Introduction

Drilling is an important technology for the exploitation of coalbed methane, shale gas, petroleum, and other mineral resources. Drill string vibration will inevitably occur during the drilling process, and the excessive vibration probably damages the drilling tool, affects the drilling efficiency, and even causes downhole accidents [1]. Therefore, it is necessary to measure the drill string vibration in the downhole in real-time. However, the traditional downhole vibration measurement methods which use cable or battery as power supplies increase the drilling costs and reduce the drilling efficiency. Hence, there are urgent needs in actual production to develop instruments that can collect the vibration energy and measure the vibration frequency in downhole.

The theory of the triboelectric nanogenerator was firstly proposed by Wang [2]. Based on the theory, researchers have developed numbers of triboelectric nanogenerator [3,4,5], which had been widely used in many fields, such as the energy collection [6,7,8,9], industrial sensors [10,11,12,13], medical equipment [14,15], leisure equipment [16,17,18,19], geological monitoring [20,21], and other industrial applications [22,23]. Therefore, the triboelectric nanogenerator brings hope for solving the problems of vibration energy collection and vibration measurement in downhole. Especially in the field of vibration energy collection, researchers had achieved a series of outstanding results, such as the 3D stack integrated triboelectric nanogenerator [24], the single-electrode three-dimensional triboelectric nanogenerator [25], the magnetically levitated triboelectric nanogenerator [26], the ferrofluid-based triboelectric-electromagnetic hybrid generator [27], the liquid metal triboelectric nanogenerator [28], the vibration-amplified triboelectric nanogenerator [29], the soft and robust spring-based triboelectric nanogenerator [30], triboelectric-piezoelectric-electromagnetic hybrid nanogenerator [31], and so on. But in the field of traditional drill string vibration measurement methods, there are just few related research results, such as the three-axis vibration sensor [32], the near-bit vibration sensor with storage function [33], the vibration measurement method using the gyroscope [34], the vibration measurement method using the decibel sensor [35], the vibration measurement method combined with machine learning [36], and so on.

The traditional drill string vibration measurement methods have limited application due to the inability to achieve self-powered sensor, and only a few publications are related to the application of the triboelectric nanogenerator in the field of geosciences, especially in the field of drill string vibration. Therefore, in order to solve the actual needs during drilling, this paper proposes a spherical triboelectric nanogenerator (or S-TENG, for short), which shows the potential to collect the downhole vibration energy to provide the power for the downhole measurement instruments and measure the vibration frequency in the self-powered model.

## 2. Design and Working Principle

### 2.1. Design Requirements

The S-TENG is installed between the downhole drill bit and the drill string, so the downhole environments have some requirements for the design of the S-TENG, namely, strong anti-interference ability, large power generation, and high reliability. The specific explanations are as follows.

Firstly, the larger the output signal amplitude, the higher the signal-to-noise ratio, and the stronger the anti-interference ability of the S-TENG. Therefore, the S-TENG should have a larger contact or electrostatic induction area to ensure a larger output signal amplitude. Secondly, the larger the contact or electrostatic induction area of the friction layer, the larger the power generation of the S-TENG. Therefore, the S-TENG should be as large as possible to increase the contact or electrostatic induction area. Thirdly, the fewer mechanical parts, the more reliable of the S-TENG. Therefore, the S-TENG should depend on its own weight instead of springs to return to the original position to reduce the use of mechanical parts. In summary, a larger-size spherical triboelectric nanogenerator is used for this paper.

**Figure 1:**
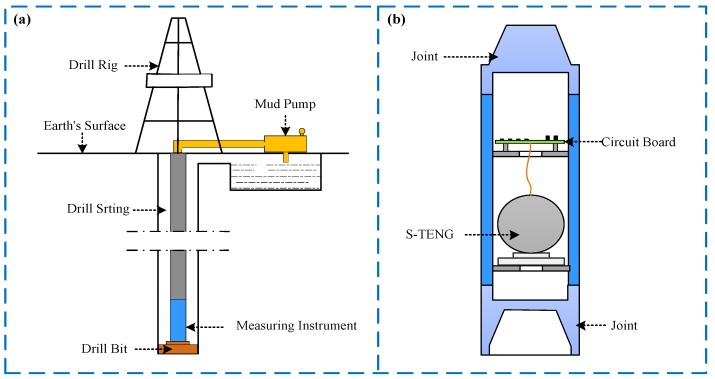
Installation location of the S-TENG (spherical triboelectric nanogenerator). (**a**) Installation location in the downhole; (**b**) Enlargement diagram of the measuring instrument.

Meanwhile, the S-TENG must be installed in a professionally designed measuring instrument with a sealing function when used in actual drilling environments (as shown in Figure 1). The properties of the measuring instrument, including the mechanical properties and the dimensions, are generally the same or similar to the drill string, and the measuring instrument is generally installed between the drill bit and the drill string. Due to the different sizes of drill string used in different drilling processes, the sizes of S-TENG under different drilling processes are also different. The larger the size, the larger the power generation, but the smaller the scope of application. Therefore, the size of the S-TENG is limited to 110 × 110 × 120 (unit mm) after careful consideration.

In addition, the vibration frequency range of the drill string is relatively large, because it is closely related to the formation conditions, well structure, drilling process parameters, and drilling tool combinations, etc. However, the drill string vibration of the ordinary low-speed drilling process is generally caused by the collision with rock and soil during the rotation of the drilling tool. As the speed of the drill string is mostly maintained at about 120 r/min in the ordinary low-speed drilling process, the basic vibration frequency generated by this is about 2 Hz. Therefore, the vibration frequency range is set to within 8 Hz, which can meet the measurement requirements of the ordinary low-speed drilling process.

### 2.2. Assemble Design and Manufacturing

**Figure 2:**
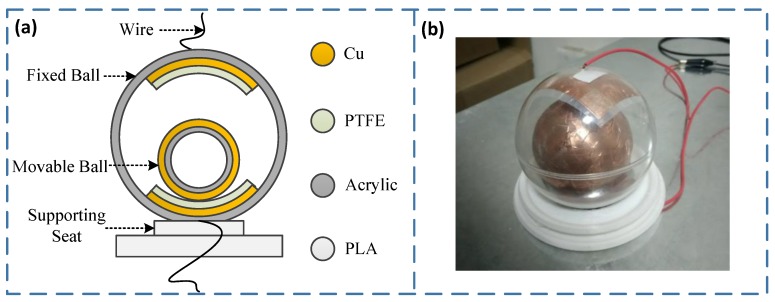
Composition of the S-TENG. (**a**) Composition schematic diagram; (**b**) A picture of the S-TENG.

As shown in Figure 2, the S-TENG is composed of a fixed ball, a movable ball, and a supporting seat. The fixed ball is fixed on the supporting seat and keeps stationary with the supporting seat, and the movable ball is embedded in the fixed ball and can move freely. Cu (Copper) and PTFE (Polytetrafluoroethylene) are pasted on the upper and lower sections of the fixed ball. PTFE is used as a friction layer to generate electric charges, and Cu is used as an electrode to derive the electric charges generated by PTFE. Cu is also attached on the surface of the movable ball and is used as both the friction layer and the electrode. 

The processing method of the S-TENG is as follows. The supporting seat is 3D printed with PLA (Polylactic acid) material, the printing temperature is 210 Celsius, the thickness of the printing layer is 0.2 mm, and the structure duty cycle is 90%. Both the fixed ball and the movable ball are made of acrylic hollow material, and the outer diameters are 100 mm and 75 mm, respectively, with a wall thickness of 3 mm for them. The thickness of the Cu (C1100, ZYTLCL Co., LTD., Dongguan, Guangdong, China) used in the fixed ball and the movable ball are both 0.05 mm, and the PTFE (CTF30, Bench Co., LTD., Suzhou, Jiangsu, China) is 0.03 mm. The sizes of PTFE and Cu pasted on the fixed ball are 70 × 70 (unit mm) and 60 × 60 (unit mm), respectively.

### 2.3. Working Principle

**Figure 3:**
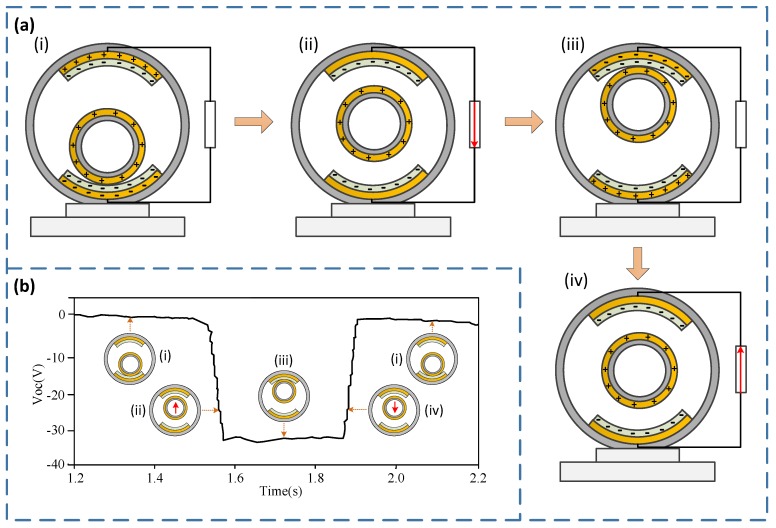
Schematic diagram of the working process of the S-TENG. (**a**) Schematic diagram of the charge transfer rule in the working process of the S-TENG; (**b**) Theoretical output voltage signal of the S-TENG.

The S-TENG is fixed inside the drill string by fixing the supporting seat. The movable ball moves up and down relative to the fixed ball when the axial vibration of the drill string occurs and generates numbers of charges due to triboelectricity and electrostatic induction. But the electrostatic induction should be the main factor, because the contact area of the two balls is very small and which can even approximate point contact. Then the vibration energy can be collected by processing the charges, and the vibration frequency can be measured by analyzing the transfer rules of the charges. Figure 3 shows the schematic diagram of the working process of the S-TENG. The working principle is further explained as follows.

The movable ball can be precharged when the S-TENG works at least once. Figure 3a-i shows the initial state of the S-TENG. The movable ball is positively charged, and the fixed ball’s lower friction layer is negatively charged because the Cu is more likely to lose electrons than the PTFE [37] under the triboelectricity and electrostatic induction. Figure 3a-ii shows the state when the vibration occurs, and the movable ball moves upward to the middle position. At this stage, the electrons flow through the external load and form the electrical current. Figure 3a-iii shows the state when the movable ball contacts the upper friction layer of the fixed ball, and the upper friction is negatively charged due to the triboelectric and electrostatic induction. Figure 3a-iv shows the state when the movable ball moves downward to the middle position, and the electrons flow through the external load reversely and form a reverse current. Figure 3a-i shows the state when the movable ball falls to the initial position, and the charges are transferred to the lower friction layer, which returns to the initial state. During the working process of the S-TENG, the theoretical output voltage signal is shown in Figure 3b.

## 3. Testing Results

Tests are divided into two parts, one is the power generation tests that collect the vibration energy, and the other is the sensing tests that measure the vibration frequency. A vibration platform which could obtain different vibration frequency at the same amplitude by adjusting the controller was used for the tests. The vibration platform is mainly composed of a simulation drill rig, a simulation drill string, and a vibration table. The basic principle of the vibration platform is that the rotary power provided by the simulation drill rig is transmitted to the simulation drill string through a cam mechanism, which causes the simulating drill string to impact the vibration table in the vertical direction. By adjusting the speed controller of the simulated drill rig, different vibration frequencies can be obtained, thereby realize the simulation of the vertical vibration. Since the contour size of the cam is fixed, the vibration amplitude is theoretically equal to it, and the amplitude is 4 mm. The S-TENG was fixed on the vibration platform, and the output signal was measured and displayed by an electrometer (6514, Keithley Co., LTD., Solon, Ohio, America). The specific test results are as follows.

### 3.1. Power Generation Performance Tests

The S-TENG shows the potential to collect the vibration energy of the downhole drill string and convert it into electrical energy in real-time. Therefore, the power generation performance of the S-TENG was tested, and the following conclusions can be obtained.

**Figure 4:**
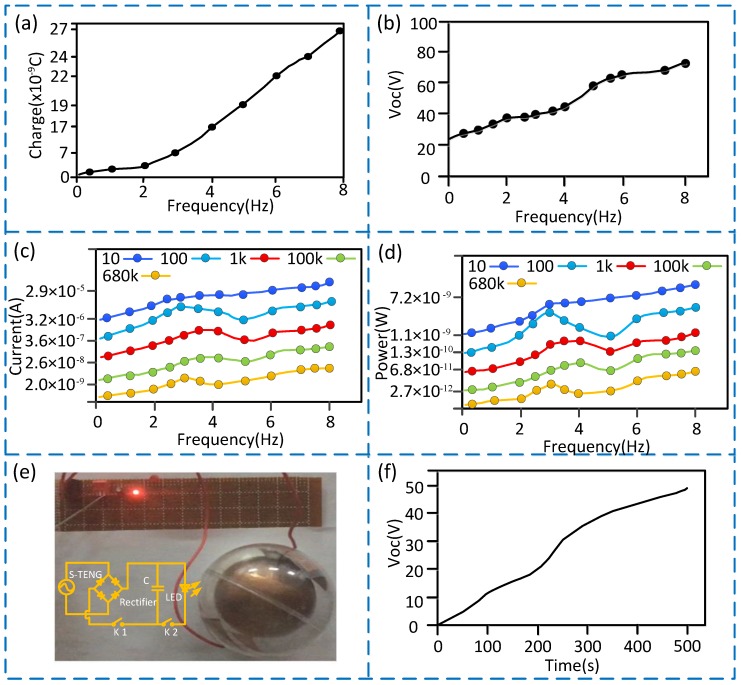
Test results of the S-TENG power generation performance. (**a**) Curve showing the variation of the transferred charges over the vibration frequency; (**b**) Curve showing the variation of the output voltage over the vibration frequency; (**c**) Curve showing the variation of the output current over the vibration frequency with different resistances; (**d**) Curve showing the variation of the output power over the vibration frequency with different resistances; (**e**) A picture showing the LED (light-emitting diode) lighted by the S-TENG; (**f**) charging curve of the capacitor (50 V, 2 μF) with the load resistance (1 k) in 8 Hz.

(1) As shown in Figure 4a–d, the output voltage, output current, and output power of the S-TENG are all the average of 1000 measurements, and they increase as the vibration frequency increases. The output voltage, output current and output power all reach the minimum value when the vibration frequency is 0.5 Hz, and the minimum voltage is 27 V, the minimum current and the minimum power are all close to 0. The output voltage, output current, and output power all reach the maximum value when the vibration frequency is 8 Hz, and the maximum voltage is 70 V, the maximum current is 3.3 × 10^−5^ and the maximum power is 10.9 × 10^−9^ W when a 10-ohm resistor is connected. In addition, the output current and output power of the S-TENG decrease with the increase of resistance value, and reach the maximum value at 10 ohms, which indicates that the power supply capability of the S-TENG is weak and needs to be further improved. (2) In order to visually show the power generation performance of the S-TENG, the output power was stored into a capacitor after processing by the circuit (as shown in Figure 4e)), with the switch k1 is closed and switch k2 is opened. Then a 0.25 W LED (light-emitting diode) was lighted when switch k2 is turned on after about 3 minutes, and the charging curve of the capacitor (50 V, 2 μF) with the load resistance (1 k) is shown in Figure 4f. Hence, if the power generation is stored for a certain period of time when numbers of S-TENG are connected in parallel, it is possible to provide intermittent power for the low-power downhole measurement instruments.

### 3.2. Sensing Performance Tests

The S-TENG shows the potential to be used as a self-powered vibration sensor to measure the vibration frequency of the downhole drill string. Therefore, the sensing performance of the S-TENG was tested in self-powered, and the following conclusions can be obtained.

**Figure 5:**
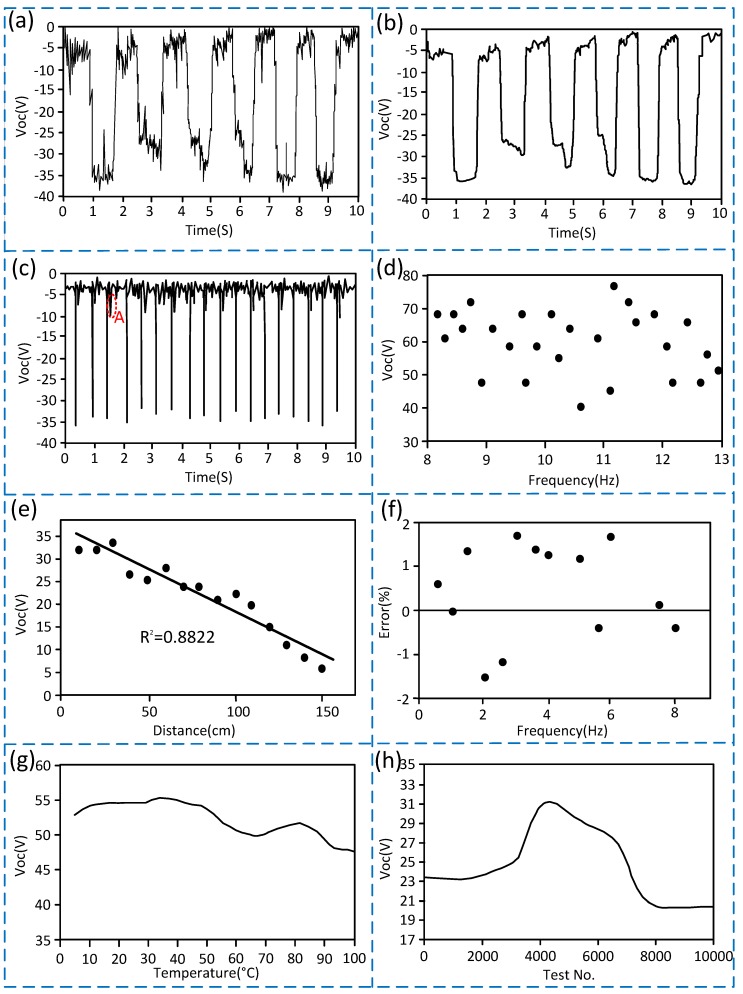
Test results of the S-TENG sensing performance. (**a**) Curve showing the output voltage of the S-TENG when the vibration frequency is 0.6 Hz (Unfiltered signal); (**b**) Curve showing the output voltage of the S-TENG when the vibration frequency is 0.6 Hz (Filtered Signal); (**c**) Curve showing the output voltage of the S-TENG when the vibration frequency is 1.8 Hz; (**d**) Scatter plot of output voltage of the S-TENG when vibration frequency exceeds 8 Hz; (**e**) Curve showing the variation of the output voltage over the distance from the vibration source when the vibration frequency is 2 Hz; (**f**) Measurement error scatter plot; (**g**) Curve showing the variation of the output voltage over the temperature when the vibration frequency is 4.5 Hz; (**h**) Curve showing the variation of the output voltage over the test times when the vibration frequency is 1 Hz.

(1) As shown in Figure 5a–c, the vibration frequency of downhole drill string corresponds to the number of voltage pulses, so the vibration frequency can be measured by the voltage pulses signal. A common low-pass filter algorithm that had been embedded into the software to process the noise signal, and the effect is shown in Figure 5a,b. Since ordinary MCU (Microprogrammed Control Unit) all have the function of pulse measurement and the trigger mode is a negative pulse trigger, the corresponding pins of the MCU can be directly connected to the S-TENG for statistics of vibration frequency. (2) As shown in Figure 4a,b and Figure 5c, the rule of the output voltage is similar to the transferred charges, the output voltage signal amplitude and the transferred charges of the S-TENG all increase as the vibration frequency increases, and the output voltage signal amplitude is larger than the noise signal. Therefore, the signal-to-noise ratio is high, and the anti-interference ability is strong. In addition, the vibration frequency and the output voltage signal are not an absolute linear function. (3) As shown in point A of Figure 5c, the output voltage signal will generate a large pulse interference when the vibration frequency exceeds about 1.8 Hz. The reason is that the movable ball will stop moving at the initial position when the measurement is completed in lower frequency vibration, but it does not stop and fluctuates near its initial position when in higher frequency vibration, which causes a large pulse interference. (4) As shown in Figure 4b and Figure 5d, the output voltage will become scattered and irregular when the vibration frequency exceeds 8 Hz, so the measurement range is 0 to 8 Hz. (5) As shown in Figure 5e, in order to research the influence of the installation distance between the S-TENG and the vibration source on the output signal, the S-TENG is fixedly mounted on the vibration platform, and the distance from the vibration source is adjustable. The results show that the output signal amplitude is inversely proportional to the distance between the S-TENG and the vibration source, and the relevance R^2^ is approximately 0.8822. The output voltage is reduced to about 5 V when the distance is greater than 150 cm, in which case the signal-to-noise ratio is unapparent. Therefore, the installation distance between the S-TENG and vibration source should be less than 150 cm and the closer the distance, the better the signal-to-noise ratio. (6) Figure 5f is a scatter plot containing the largest error after statistical analysis of the measurement data. The total measurement data is 500 groups, and each group is the average value after 3 minutes of measurement. We can obtain that the measurement error at 0 to 8 Hz is less than 2%. (7) As shown in Figure 5g, the minimum voltage value of the S-TENG is still higher than 45 V when the temperature changes within 0 to 100 degrees Celsius, which is much larger than the amplitude of the noise signal. Therefore, the S-TENG can be used when the good temperature does not exceed 100 degrees Celsius. (8) Figure 5h is the test results when the vibration frequency is 1 Hz, and the test time for each test No. is 20 seconds. The output voltage gradually increases to a maximum of 31 V when the test No. is less than 4500, and it will then gradually decrease to 20 V and remains stable when the test No. (number) is between 4500 and 10,000. Even if the output voltage is reduced to 20 V when the test times reaches 10,000, the voltage value of 20 V still has a high signal-to-noise ratio and anti-interference ability because the MCU with TTL (Transistor-Transistor Logic) voltage standard can still recognize it as a high-level signal and detect it as long as the output signal is greater than 5 V. So, it shows that the S-TENG has high reliability.

## 4. Conclusions and Discussions

(1) The S-TENG shows the potential to collect the vibration energy of the downhole drill string, and the output signal increases as the vibration frequency increases. The output voltage, output current, and output power all reach the maximum value when the vibration frequency is 8 Hz, and the maximum value is 70 V, 3.3 × 10^−5^ and 10.9 × 10^−9^ W, respectively. Hence, if the power generation is stored for a certain period of time when numbers of S-TENG are connected in parallel, it is possible to provide intermittent power for the low-power downhole measurement instruments. But the power generation of a single S-TENG is relatively small, and it is far from the goal of real-time power supplies for the downhole measurement instruments. Therefore, the next research will begin through the generation of nanowires, the surface characteristics of nanomaterials, and the synthesis of new nanomaterials to further increase the power generation performance of a single S-TENG.

(2) The S-TENG shows the potential to measure the vibration frequency of downhole drill string. The measurement range varies from 0 to 8 Hz, the measurement error is less than 2%, the applicable working environment temperature is below 100 degrees Celsius, the signal-to-noise ratio is high, and the anti-interference ability is strong. In addition, the output signal amplitude is inversely proportional to the distance between the S-TENG and the vibration source, and the signal-to-noise ratio is unapparent when the distance is more than 150 cm. As the downhole vibration is mainly generated by the collision between the drill bit, and the rock, the S-TENG should be installed near the drill bit, and the installation distance should be less than 150 cm. The measurement range is small, and it is only suitable for the low-speed drilling processes. Therefore, the next research is to change the structure to increase the measurement range of the S-TENG further.
